# The Effect of Visual Mnemonics and the Presentation of Character Pairs on Learning Visually Similar Characters for Chinese-As-Second-Language Learners

**DOI:** 10.3389/fpsyg.2022.783898

**Published:** 2022-05-09

**Authors:** Li-Yun Chang, Yuan-Yuan Tang, Chia-Yun Lee, Hsueh-Chih Chen

**Affiliations:** ^1^Department of Chinese as a Second Language, College of International Studies and Social Sciences, National Taiwan Normal University, Taipei, Taiwan; ^2^Department of Educational Psychology and Counseling, College of Education, National Taiwan Normal University, Taipei, Taiwan

**Keywords:** Chinese-as-second/foreign-language (CSL/CFL) learning, Chinese orthographic learning, material presentation, visual mnemonics, visually similar characters

## Abstract

This study investigates the effects of visual mnemonics and the methods of presenting learning materials on learning visually similar characters for Chinese-as-second-language (CSL) learners. In supporting CSL learners to build robust orthographic representations in Chinese, addressing the challenges of visual similarity of characters (e.g., 理 and 埋) is an important issue. Based on prior research on perceptual learning, we tested three strategies that differ in the extent to which they promote interrelated attention to the form and meaning of characters: (1) Stroke Sequence, a *form-emphasis* strategy, (2) Key-images, a *form* + *meaning* strategy utilizing visual code, (3) Pithy Formulas with Key-images, a *form* + *meaning* strategy combining visual and verbal codes. A pretest–posttest equivalent-group design was adopted. The independent variables were the learning strategy, the method of presenting character pairs (visually similar vs. dissimilar), and testing time. The dependent variables were learners’ proportions of accurate responses to reading and writing Chinese characters through a posttest (immediately performed after learning) and a delayed posttest (1 week after learning); a learner experience survey was also administered to investigate learners’ opinions on each strategy. Sixty-six non-beginning learners of Chinese participated; they were randomly assigned to one of the two groups in which participants learned ten characters *via* the three strategies, respectively, differing between whether the characters were presented in similar pairs or dissimilar pairs. Data were analyzed *via* three-way ANCOVAs. The Pithy Formulas with Key-images and the Key-images generally yielded higher writing accuracy than Stroke Sequence immediately after learning. Notably, the advantage of the Pithy Formulas with Key-images (verbal and visual) over the Key-images (visual) on writing was specific to the participants that learned with visually similar pairs rather than those that learned with dissimilar pairs. All strategies were effective for reading, yet learners’ experience ratings favored the two *form* + *meaning* strategies over the strategy that focused primarily on *form.* Suggestions for future research and pedagogical implications on learning visually similar characters were offered.

## Introduction

Chinese writing contains the most complicated graphs in the world (Chang et al., 2018), raising the difficulty in character learning. The various combinations of multi-layered orthographic units of Chinese graphs (e.g., strokes, radicals, characters, and structures) conspire to present significant challenges to Chinese-as-second/foreign-language (CSL/CFL) learners (DeFrancis, 1989; Perfetti et al., 2005; Shen, 2005, 2013). CSL/CFL learners often regard Chinese as the most difficult language to learn (Yu, 2012). To become a skilled reader of Chinese, however, the learners are expected to master 3,000 frequent characters (Ma et al., 2017) to achieve automatic recognition and proficiency in character writing. In accumulating character learning up to the thousands range, it is inevitable that the learners would encounter visually similar characters, which look alike orthographically (Perfetti, 1999).

Prior research has defined visual similarity as the degree to which orthographic features of Chinese characters overlap (cf. Yeh et al., 1997; Yeh and Li, 2002; Liu et al., 2011). Specifically, a feature-based similarity analysis revealed that two characters with the same structure, one common radical, and stroke discrepancies in other radicals represent a highly visually similar pair (Yeh and Li, 2002). For instance, the forms of 埋 and 理 seem to be almost the same at the first glance. However, 埋 (mái), consisting of the components 土 and 里, means “to bury,” whereas 理 (li), consisting of 王 and 里, refers to “management.” Such visual similarity has accounted for 75% errors in 4,100 incorrectly written characters in a computational linguistics study (Liu et al., 2009). Indeed, interference from the visual similarity to character learning is also evident in writing errors made by CSL/CFL learners (e.g., Teng et al., 2008; Qin, 2014; Gan, 2020). In a larger analysis on 4,305 written responses from 144 CSL learners with different Chinese proficiencies (Teng et al., 2008), errors at the stroke level were found to account for the most variance in accuracy rate; among the variance of stroke errors, over 50% belong to the omission and addition error type. Moreover, the omission and addition errors were found to be the most common error types in a systematic analysis on beginning-to-intermediate CFL learners’ writing errors in workbooks collected over the span of 1 year (Gan, 2020). The results of the studies mentioned above on writing errors may reflect that visual similarity of characters creates a huge learning hurdle for CSL/CFL learners.

Based on learning theories investigating how learners perceive, process, and maintain knowledge in learning, Chou (2009a) suggested that both objective and subjective factors may influence learning. The objective factors include, but are not limited to, learning targets (e.g., visually similar characters), learning strategies, and methods of presenting learning materials. The subjective factors consist of motivational and affective aspects (e.g., enjoyment in using strategies, the usefulness of strategies, the ease of using strategies, and learners’ willingness to use strategies in the future). Chou (2009b) proposed that “learning by visual mnemonics” is the most effective strategy for learning visually similar characters. However, scientific examination on these proposals remains scarce. To date, there have been no empirical investigations performed with the aim of supporting CSL/CFL learners to overcome the hurdles of distinguishing visually similar characters. Further research is necessary, as the first languages of the majority of these learners do not contain such complex orthography.

To fill this gap, this study developed visual mnemonics that differ in the extent to which they promote interrelated attention to the form and meaning of Chinese characters. These mnemonics were developed based on the dual-coding theory, which postulates that information coded in both verbal and visual codes has additive effects on memory (Paivio, 1986), and the Elaboration theory, which proposes that learning materials should be organized from simple to complex and further into meaningful context (Reigeluth, 1999), to facilitate complex compound learning. We sought to elucidate how different strategies and methods of presenting characters may influence the reading and writing of CSL/CFL learners with basic knowledge of Chinese in terms of mastering visually similar characters.

In what follows, we introduce the difficulties of learning Chinese characters (1.1), empirical research on the learning of visually similar graphs across writing systems and within the Chinese writing system (1.2), learning strategies and the use of visual mnemonics in supporting character learning (1.3), and the present study (1.4).

### The Difficulties of Learning Chinese Characters

The Chinese writing system is logographic in nature, given that its written symbols represent lexical morphemes instead of individual phonemes (Perfetti and Dunlap, 2008). Learning to read involves interconnections among three lexical constituents – form (orthography), sound (phonology), and meaning (semantics), based on the Lexical Constituency Model (Perfetti et al., 2005). Such interconnections are complex for Chinese characters because a syllable (sound) is associated with many different morphemes (meaning) and different characters (form). On average, one syllable is shared by eleven characters with different meanings (Language and Teaching Institute of Beijing Linguistic College, 1986). Given this characteristic of the form-sound correspondence, using the sound to mediate the link between meaning and form is difficult when learning to read Chinese (Perfetti et al., 2005; Shen, 2005, 2013).

However, decoding (e.g., recognizing characters) and comprehension are both critical skills in learning to read (Hoover and Gough, 1990). This simple view of reading is a profound notion in Chinese-as-first-language (L1) studies (e.g., Ho et al., 2012) and second language research (e.g., Verhoeven and van Leeuwe, 2012). Based on this view, mastering Chinese characters, measured by automatic reading and proficiency in writing, lays a foundation and pervades subsequent learning of the Chinese language. Nevertheless, the task of learning characters is difficult because there are tens of strokes, hundreds of radicals/components, various structures/positions, and thousands of characters to be memorized (Chen et al., 2011; Wang, 2011; Ma et al., 2017). Thus, supporting learners of Chinese to develop robust orthographic representations in reading and writing characters is important (Koedinger et al., 2012).

To overcome the difficulties of learning Chinese characters, developmental research on CSL/CFL learners’ orthographic awareness has shown that learners’ sensitivity to components with different functions (e.g., semantic or phonetic) may help them to learn characters (e.g., Leck et al., 1995; Shen, 2005, 2013; Shen and Ke, 2007; Kuo et al., 2015). For instance, Kuo et al. (2015) reported that CSL learners with a novice-intermediate proficiency level of ACTFL (American Council on the Teaching of Foreign Languages, 2012) in reading and writing Chinese can decode compound characters into informative semantic components, showing radical awareness during novel character learning. The compound characters, which are usually semantic-phonetic compounds with one semantic radical signifying the meaning and the other radicals suggesting the sound of the compound, account for 90% of Chinese characters (Shen, 2005). Moreover, for compound characters, meaning cues from the semantic radicals are relatively more reliable than the pronunciation cues from the phonetic radicals (Ho et al., 2003). For instance, in 6,097 characters (Chen et al., 2011), there are 260 characters with the semantic radical “扌.” In these 260 characters, 248 characters are related to the meaning of “hand” or “motion”; the transparency of semantic radical “扌” is 95% (248/260). On the contrary, the accuracy of the pronunciation of an ideophonetic compound character from its phonetic radical is approximately 40% (Shu et al., 2003). These analyses were in line with CSL/CFL research, suggesting that learners with intermediate-high proficiency predominantly use semantic radicals, over phonetic radicals, in learning compound characters (Taft and Chung, 1999; Williams, 2013).

Taken together, within the complex interconnections among forms, sound, and meanings of characters, forms link more reliably to meaning-bearing morphemes (Perfetti et al., 2005). Given this relatively reliable association, this study aims to investigate the plausibility of strengthening the form-meaning links to help CSL/CFL learners overcome the hurdles described above and more effectively differentiate between visually similar characters, which are further described in the next section.

### Empirical Research Within and Across Writing Systems on the Learning of Visually Similar Graphs

Visual similarity of graphs may influence the development of high-quality orthographic representations (Perfetti, 2007), thus contributing to difficulty in learning to read within (Hirshorn and Harris, 2022) and across different writing systems (Chang et al., 2018). Although the number of graphs in alphabets (i.e., single letters or letter combinations), typically ranging from 20 to 45 (Verhoeven and Perfetti, 2021), is much lower than Chinese, visual similarity among alphabetic letters plays a prominent role in the initial stage of learning to read: the perceptual learning of graph forms.

Gibson’s (1969) theory of perceptual learning, investigating over 70 years of studies with participants of all ages (for review, see Adolph and Kretch, 2015), asserts that learning to read is fundamentally learning to detect features that specify different graphs through higher order relations (Gibson and Levin, 1975; Gibson, 1991). To examine the higher-order relations, a series of studies manipulated the methods of presenting English letters as learning materials (e.g., Samuels, 1969; Williams and Ackerman, 1971), including appearance of letters (visually similar or dissimilar letter pairs; i.e., b/d vs. b/s) and the method of presentation (simultaneously or successively). With 88 first graders as participants, Williams and Ackerman, 1971 showed that presenting highly similar letters simultaneously led to the worst outcomes. Contrary to the findings, Samuels (1969) recruited 60 kindergarteners and randomly assigned them to two groups (simultaneous vs. successive). Using highly similar letters as materials, Samuels (1969) reported that the simultaneous group outperformed the successive group in discriminating and identifying the correct letter forms. While findings in English research are mixed, these studies at least suggested that the method of presenting learning materials matters.

Scientific studies on learning visually similar Chinese characters are scarce and rarely focus on how methods of presenting characters influence learners’ reading and writing. To the best of our knowledge, only two journal articles have adopted rigorous pretests and posttests between subject research designs and reported comparisons on learners’ performance in learning visually similar and dissimilar characters (Lin, 1997, 1998). In both studies, characters were taught either by visual similarity or visual dissimilarity. Learning performance was measured *via* recognition and writing tasks. Findings revealed that first graders with high or mid-range achievement of Chinese benefited more from presenting characters with visual dissimilarity (Lin, 1997). However, for students with low Chinese achievement, presenting characters with similarity yielded better writing than presenting characters with dissimilarity (Lin, 1998). No presentation difference was found in recognition. Although these studies showed that methods of presenting learning materials affect reading and writing, such findings came from L1 first graders with low Chinese achievement, and the effects of material presentation for CSL/CFL learners remain unclear. The present study aims to bridge this gap by adopting a rigorous pretest–posttest equivalent-group design with non-beginning learners of Chinese.

Meanwhile, visual similarity between characters caused by stroke discrepancies represents a significant challenge of orthographic learning for learners who have acquired basic knowledge of Chinese characters, including L1 learners. Chou (2009a) reviewed 94 journal papers on L1 learners’ character writing errors and also concluded that additive or substrative stroke is one of the main reasons for writing errors. These errors may keep recurring, resulting in further confusion in distinguishing visually similar characters from one another if the learners are not made aware of the differences in strokes *via* explicit instructions. Therefore, Chou (2009a) integrated perspectives from learning theories and Grammatology, proposing seven guidelines for teaching visually similar characters (e.g., learning characters by visual mnemonics, such as images, memorizing characters by pithy formulas, and reviewing characters by characters in a group). However, whether these guidelines have empirical implications for CSL/CFL learners’ learning visually similar characters remains to be investigated. Therefore, this study attempts to shed light on the beneficial effects of incorporating visual mnemonics in CSL/CFL character learning.

### Using Visual Mnemonics as a Learning Strategy to Support Learning for Chinese-as-Second/Foreign-Language Learners

Visual mnemonics provide a learning strategy to strengthen memory traces of orthographic and semantic constituents, in acknowledging the relatively reliable connection between form and meaning in Chinese characters. A visual mnemonic is a learning technique used to aid the association of the to-be-memorized information (e.g., forms and meanings) with mediators (e.g., imagery to represent meaning), which are more accessible to provide better retention and retrieval for learners. The power of mnemonics has been widely acknowledged in language education (for reviews, see Levin, 1993; Mohammad and Ketabi, 2011), even in Chinese (e.g., Kuo and Hooper, 2004; Shen, 2010; Wang, 2014; Packard, 2017). For instance, Chou (2009b) advocated that “vocabulary by visual mnemonics” works the best among her guidelines (Chou, 2009a) for teaching visually similar characters.

By leveraging visual mnemonics and characteristics of Chinese orthography, Chen et al. (2012) put forward a three-stage character-based instructional (TCI) framework to support character learning. The TCI framework is orthography oriented; given the different orthographic features of characters, learning can be facilitated with different strategies. The strategies are broadly categorized into three stages: (1) logographic character learning with Key-images, (2) component-deriving character learning with characters in a group, and (3) complex character learning with Pithy Formulas and Key-images. The effectiveness of the TCI, especially the Key-images for learning logographic characters, has been demonstrated in laboratories (e.g., Chen et al., 2013; Chang et al., 2019) and classrooms (e.g., Lin et al., 2021; Tsai et al., 2021) in recent years. However, the effects of Key-images on learning compound characters and the effects of combining Pithy Formulas with Key-images on learning visually similar characters remain to be investigated.

Key-images and Pithy Formulas are developed based on cognitive theories, namely the dual-coding theory (Paivio, 1986, 1990, 2006) and the elaboration theory (Reigeluth, 1999). The dual-coding theory postulates that human learning operates with two subsystems (or “codes”) of mental representation: verbal and non-verbal (i.e., visual). The visual code refers to mental imagery of learning targets, and the verbal code indicates linguistic features, which help learners to comprehend the meaning of targets; although functioning independently, these two codes can have synergic effects on recall (for reviews, see Clark and Paivio, 1991). For the visual code, Key-images in the TCI (Chen et al., 2012) are visual imagery deliberately designed to bind an image of form with an image of target learning materials, thus promoting learners’ attention to *form* + *meaning* to characters. That is, a Key-image is designed to be both visually like the form of a character and highly associable to its meaning. Such design has been applied to 445 logographic characters, available in CSL/CFL textbooks (Chen and Lin, 2015a,b; Chen et al., 2021). As for the verbal code, pithy formulas are brief sentences but full of information about the learning target. Take the character “碧” for example, it can be decomposed into components “王, 白, and 石.” The pithy formula for “碧 (jade)” would be “‘王(Wang)’先生和‘白(Bai)’小姐坐在‘石(rock)’頭上” (*Mr. Wang and Miss Bai are sitting on a rock.*).

The “碧” example is also an instantiation of elaboration, a strategy to organize learning content into meaningful context, helping learners to construct knowledge in their minds (Reigeluth and Stein, 1983; Reigeluth, 1999). The Pithy Formulas in the TCI (Chen et al., 2012) is more informative than the previous one. For instance, the character “評” can be decomposed into components “言” and “平”; its Pithy formulas are “The judges should say (言) fair (平) evaluations (評) to the athletes.” In this case, not only are the meaning of each component and their compositions mentioned, but the meaning of the whole character is explicitly shown. That is, such verbal code elaborates the part-whole relationship in components characters (Nguyen et al., 2017). Moreover, in combining Pithy Formulas (verbal code) and Key-images (visual code), the synergic effects of dual codes may enhance memory consolidation and further stabilize form-meaning association in the learning of visually similar characters. These effects merit further investigations.

In accumulating character learning experiences, adult CSL/CFL learners may adopt various learning strategies (e.g., Ke, 1998; Shen, 2005; Winke and Abbuhl, 2007; Lam, 2011; Sung, 2012). In a comprehensive investigation with a semi-structured survey and open-ended questions to CSL learners at different levels, Shen (2005) identified 59 character-learning strategies and reported that the most commonly used one is the orthographic-knowledge-based strategy. The author interpreted this finding based on the logographic nature of Chinese (i.e., the various orthographic features and the absence of reliable form-sound correspondence); the former may encourage form-emphasis strategies (e.g., focusing on stroke sequence), and the latter may make use of *form* + *meaning* association strategies. Stroke sequence, a *form-emphasis* strategy, presents how a character is composed by strokes. Stroke sequence is a commonly used strategy perhaps because students usually see stroke sequence in textbooks along with character copying or writing exercises (Jin, 2006). In some discussions on whether reading depends on writing (Tan et al., 2005) or not (Bi et al., 2009), stroke sequence as a learning strategy for supporting orthographic learning is often mentioned because it probes the relationship between reading and writing (Xu et al., 2013). However, although this rote strategy is frequently used in Chinese language instruction, CSL learners often see the process as uninteresting, and, thus, they hope for a “pen-less” experience (Xu and Jen, 2005). Taken together with the previously reviewed learning strategies, the present study investigates the effects of *form emphasis* (e.g., Stroke Sequence) and *form* + *meaning* congruence (e.g., Pithy Formulas and Key-images), with a consideration on learners’ affective opinions on each strategy.

### The Present Study

Prior to the present work, previous studies had been performed (Chang et al., 2019; Lin et al., 2021) with Key-images in learning logographic characters for adult beginning learners of Chinese, but there is a lack of empirical studies done with CSL/CFL learners with a novice-intermediate proficiency level. Therefore, we implemented a pretest–posttest laboratory learning experiment, making this study the first attempt to apply Key-images to learning visually similar characters and the first to explore the degree to which the method of presentation may influence learning visually similar characters for non-beginning learners of Chinese.

The purpose of this study was to examine effects of three learning strategies (Pithy Formulas with Key-images, Key-images, and Stroke Sequence) and two methods of presenting learning materials (visually similar pairs and dissimilar pairs) in reading and writing visually similar characters for non-beginning CSL learners. Based on the literature review, we asked the following research questions:

(1) What are the effects of learning strategies (Pithy Formulas with Key-images, Key-images, and Stroke Sequence) on learning to read and write visually similar characters?
(2) What are the effects of presenting materials (similar vs. dissimilar pairs) on learning to read and write visually similar characters?
(3) Is there an interaction between the three learning strategies and two methods of presentation in reading and writing visually similar characters over time (immediately after learning and 1 week after learning)?
(4) How do CSL learners perceive their learning experience with these strategies in terms of enjoyment, usefulness, ease of use, and willingness to use in the future?

## Materials and Methods

### Design

A 3 (learning strategy)- × -2 (method of material presentation)- × -2 (testing time) mixed design was carried out with learning strategies (Pithy Formulas with Key-images, Key-images, and Stroke Sequence) and testing time (immediate posttest and delayed posttest) as two within-subject variables; the method of presentation (similar and dissimilar groups) as a between-subject variable. The dependent variables were the accuracy of character recognition and writing, with additional measures of learner experience ratings on the three learning strategies.

### Participants

We determined a sample size of 66 by conducting a priori power analysis for sample size estimation using G*Power 3 (Faul et al., 2009) for an *F*-test at a 5% type one error level, 80% power, and Cohen’s *f* = 0.4 effect size, as assessed by a pilot study. We followed the methodology implemented in Chang et al. (2019), and we interviewed the participants in the pilot to determine the presentation duration, ensuring our participants had sufficient time to absorb the content. The 66 participants (26 females) were students who studied Chinese in Taiwan. They studied traditional characters and reported that they had been using *Hanyu Pinyin*, a phonetic transcription system of spoken Mandarin Chinese. Their ages ranged from 18 to 43 years old (*M* = 28, *SD* = 7.12). Twenty-two of them came from the United States, 22 from Europe, and 22 from Asia. They reported the following background information, as assessed by a language history questionnaire (Tokowicz et al., 2004): (1) have an above-A2 level of Chinese according to the Common European Framework of Reference for Languages (CEFR), (2) have lived in Taiwan for more than 3 months, (3) right-handed, (4) have normal or corrected-to-normal vision, (5) have normal hearing, and (6) have no history of having any learning disorders. They received monetary compensation for their participation. This study was approved by the Institutional Review Board (IRB) of a university in northern Taiwan.

### Stimuli

Thirty traditional Chinese characters were selected from the Chinese Orthography Database (Chen et al., 2011). Given that visually similar characters were of the focus in this study, these 30 characters were selected based on the following considerations: (1) all are compound characters; (2) all composing components have corresponding Key-images; (3) the visually similar pairs belong to the type of additional-or-subtractive strokes to enhance visual similarity. For each similar pair, the characters were matched by the following properties: (1) structure (left-right, or top-down), (2) number of components (*M* = 2), (3) number of strokes (*M* = 11) and the difference of stroke counts within a pair all below 3, and (4) frequency of English translations (Brysbaert and New, 2009). As for how these characters were paired together, in the similar group, two visually similar characters were always in a pair, whereas in the dissimilar group, two visually similar characters were never paired with each other. For a careful design, in the dissimilar group, we ensured that no identical radical was shared by the two characters in each pair, and we minimized the difference in stroke counts within each pair (*M* =1.60, *SD* = 1.40).

Table 1 provides a sample organization of the learning materials in terms of their similar/dissimilar presentations; Appendix 1 (see Supplementary Material) illustrates how these materials are presented in the form of Pithy Formulas with Key-images.

**TABLE 1:** Pairs of learning materials (30 characters; 15 pairs in each group) between both groups (i.e., similar vs. dissimilar groups).

Characters learned in the similar group			Characters learned the dissimilar group		
Block 1	Block 2	Block 3	Block 1	Block 2	Block 3
埋理	塊瑰	忡怏	埋貢	賈間	鈦話
責貢	買賈	查杳	稚討	棵詳	李怏
書畫	問間	鈦鈸	畫責	塊評	詁查
稚椎	稞棵	話詁	計理	買棵	忡杳
計討	評詳	季李	書稚	瑰問	鈸季

### Measurement

The learning measures included a character writing task assessing productive form representation and two recognition tasks assessing form-meaning (Chinese to English) and meaning-form connections (English to Chinese), respectively. In addition to the learning measures, a learner experience survey based on prior research (Chang et al., 2019) was given to assess the participants’ attitudes toward each given strategy. These measures are described below; instructions to participants and examples for each learning measure are provided in Appendix 2 (see Supplementary Material).

#### Character Writing

The writing task asked the participants to write a character from memory based on a given prompt of English words. They were encouraged to try their best in completing the task by being promised partial credit for their responses. Responses were scored by two schemes – an all-or-none scheme (character scoring) and a continuous scheme (stroke scoring). Stroke scoring is a partial-credit-given scheme; the score of a character is a proportion of correct strokes produced (i.e., the denominator is the character’s total number of strokes, and the numerator is the number of correct strokes in the written response). In contrast, character scoring is a strict scheme in which credit (Score 1) is given only for an exact reproduction of the whole character, while all other responses are scored 0. Scores from these two schemes may reflect the extent to which learners can recall and reproduce the character forms. Previous studies supported the higher sensitivity of using stroke scoring relative to character scoring (e.g., Chang et al., 2014, 2019) to assess the degree of correctness of learners’ orthographic representation. For instance, consider the scores for the character meaning “evaluation” (評). The written response 訐 would be scored 0 in character scoring while coded as 83.33% (10/12) in stroke scoring.

Given that partial-credit-given scoring might involve different judgments on each correctly placed stroke, one researcher scored the entire set of written responses and a second researcher independently scored one-third of the responses. These responses were selected by stratified sampling, i.e., from the pre-test, the immediate posttest, and the delayed posttest; one third of the written responses were randomly sampled. Pearson product moment correlation was performed on the cases scored by the two researchers. Inter-rater reliability in stroke scoring was 99.%, and inter-rater reliability in character scoring was 100%.

#### Character Recognition (Chinese to English and English to Chinese)

The recognition task included two subtasks: Chinese to English and English to Chinese, and a computerized multiple-choice format was adopted for both subtasks. For the Chinese-to-English recognition task, thirty characters were presented in a random order on a screen. For each Chinese character, four meanings (in English) were presented, including the correct meaning and three distractor meanings that had been paired with different characters from the same block. The participants were instructed to choose the correct meaning and then proceeded at their own paces. For the English-to-Chinese recognition task, it was particularly designed to assess the participants’ orthographic representation of visually similar characters, i.e., the ability to differentiate one character from its visually similar counterpart. This task reversed the direction of recognition by showing English words (in a random sequence) and asking the participants to choose the corresponding character. Each English word had four candidates, including the correct character, the character that was visually similar to the correct one, and two other characters that had been paired with different meanings from the same block. For both tasks, the accuracy rate was calculated by dividing each participant’s correctly responded items by the total number of items (i.e., 30) and multiplying the result by 100.

#### Learner Experience Measures

The learner experience ratings assessed the participants’ opinions of each learning strategy in four aspects: the level of enjoyment in using the strategy, the usefulness of the strategy, the ease of using the strategy, and their willingness to use the strategy in the future. These aspects were based on the Technology Acceptance Model (Davis, 1989), the most widely used framework for predicting an individual’s likelihood to accept new technology. We switched the idea of technology to learning strategy in this study, given that all learning was carried out with computers, and we believe that it is promising to combine the most accepted strategy with technology in our next project.

The participants’ responses were made on a seven-point Likert-type scale (1 = absolutely negative, to 7 = absolutely positive). There were twelve questions in total, four questions for each strategy, and the following are examples of questions regarding use of the Key-images strategy.

(1) Enjoyment of use: Please rate, from 1 to 7 (least to most), how much you *enjoyed* using the Key-images to learn Chinese characters.

(2) Usefulness: Please rate, from 1 to 7 (least to most), how *useful* you found the Key-images to learn Chinese characters.

(3) Ease of use: Please rate, from 1 to 7 (least to most), how *easy* you found the Key-images to learn Chinese characters.

(4) Willingness to use: Please rate, from 1 to 7 (least to most), how *likely* you feel you would use the Key-images to learn Chinese characters in the future.

In this study, Cronbach’s Alpha was used to test the internal consistency of the measures. The coefficient was 0.73, indicating reasonable reliability (De Vellis, 2003).

### Procedure

The procedure of the study consisted of a pretest, a learning session, a posttest (immediately after learning), and a delayed posttest (1 week after learning). All were administrated in a one-on-one fashion in an experimental lab with assistance from trained researchers specialized in teaching Chinese as a second language. Additionally, all learning measures were introduced with a practice example to make sure that the participants understood the instructions of the tasks.

Before the learning session, each participant was asked to complete the pretest as described in the measurement; all pretests shared the same form as used in the posttest and the delayed posttest. Next, the participants were randomly assigned to either the group that learned with similar pairs (the similar group), or the group that learned with dissimilar pairs (the dissimilar group). Both groups learned the same 30 characters (in pairs), while the similar group encountered 15 pairs composed of visually similar characters, and the dissimilar group encountered 15 pairs, consisting of visually dissimilar characters.

For the learning session, there were 3 blocks; a Latin square design was adopted to balance the order of the strategy and the order of the pairs. In each block, the participant used one strategy to learn 5 pairs; within each block, the sequence of pairs was randomized. Thus, all the participants experienced all three strategies in learning different character pairs (30 characters in total). Table 2 shows the Latin square design and the schedule for these participants. The numbers represent the participants’ ID.

**TABLE 2:** An experiment schedule and Latin square design used for balancing the order of learning strategies.

Pretest	Participants (*N* = 66)		Learning blocks			Post-test	Delayed posttest
	The similar group (*n* = 33)	The dissimilar group (*n* = 33)					
	1/4/7/10/13/16/19/22/25/28/31	1/4/7/10/13/16/19/22/25/28/31	P	K	S		
	2/5/8/11/14/17/20/23/26/29/32	2/5/8/11/14/17/20/23/26/29/32	S	P	K		
	3/6/9/12/15/18/21/24/27/30/33	3/6/9/12/15/18/21/24/27/30/33	K	S	P		

*P, Pithy Formulas with key-images; K, key-images; and S, stroke sequence.*

#### Pretest

A character writing task and two recognition tasks were administered to assess individual participants’ prior knowledge of the target characters. For participation eligibility, only when the participants wrote no more than three characters out of 30 characters in the character writing task could they proceed to the recognition tasks. Nine participants were excluded due to their writing accuracy rates being higher than 10%. This criterion was set by consulting prior research (Chang et al., 2014), while all accuracy rates in the pretests, including writing and recognition, were collected to track the participants’ learning trajectories compared with immediate and delayed post-tests. Table 3 shows the descriptive statistics (means and standard deviations) of all pretests.

**TABLE 3:** Descriptive statistics (*M*, standard deviation, and the adjusted *M*) of the two groups for all learning measures over time (*N* = 66).

		Group learned with similar pairs (*n* = 33)					Group learned with dissimilar pairs (*n* = 33)					
Measure	Strategy	Pretest	Immediate posttest	Delayed posttest	Adjusted *M*_*immediate*_	Adjusted *M*_*post*_	Pretest	Immediate posttest	Delayed posttest	Adjusted *M*_*immediate*_	Adjusted *M*_*post*_	Co-variates in the ANCOVA
Character writing (stroke scoring)	*P*	0.07 (0.09)	0.54 (0.24)	0.33 (0.20)	0.55	0.34	0.10 (0.12)	0.57 (0.28)	0.47 (0.23)	0.56	0.46	0.087
	*K*	0.08 (0.10)	0.43 (0.28)	0.32 (0.24)	0.45	0.33	0.09 (0.10)	0.56 (0.28)	0.40 (0.25)	0.55	0.40	
	*S*	0.09 (0.10)	0.41 (0.26)	0.27 (0.19)	0.43	0.28	0.09 (0.11)	0.48 (0.29)	0.40 (0.26)	0.47	0.39	
Character writing (character scoring)	*P*	0.04 (0.06)	0.34 (0.23)	0.17 (0.18)	0.35	0.18	0.05 (0.09)	0.39 (0.29)	0.26 (0.22)	0.38	0.25	0.041
	*K*	0.03 (0.06)	0.26 (0.26)	0.13 (0.15)	0.27	0.14	0.03 (0.06)	0.33 (0.27)	0.25 (0.24)	0.32	0.25	
	*S*	0.05 (0.07)	0.24 (0.23)	0.15 (0.15)	0.25	0.16	0.05 (0.07)	0.29 (0.25)	0.23 (0.21)	0.28	0.22	
Recognition (Chinese-to-English)	*P*	0.46 (0.18)	0.83 (0.16)	0.79 (0.19)	0.84	0.80	0.56 (0.22)	0.81 (0.15)	0.82 (0.16)	0.79	0.81	0.497
	*K*	0.43 (0.19)	0.84 (0.16)	0.78 (0.17)	0.86	0.79	0.51 (0.19)	0.81 (0.15)	0.77 (0.16)	0.78	0.77	
	*S*	0.48 (0.19)	0.78 (0.17)	0.76 (0.18)	0.79	0.77	0.55 (0.25)	0.82 (0.16)	0.82 (0.17)	0.80	0.81	
Recognition (English-to-Chinese)	*P*	0.49 (0.22)	0.77 (0.24)	0.70 (0.24)	0.80	0.73	0.53 (0.28)	0.75 (0.23)	0.71 (0.24)	0.71	0.68	0.494
	*K*	0.42 (0.23)	0.73 (0.21)	0.67 (0.23)	0.75	0.70	0.50 (0.20)	0.79 (0.22)	0.71 (0.25)	0.76	0.68	
	*S*	0.45 (0.26)	0.76 (0.23)	0.70 (0.24)	0.78	0.72	0.59 (0.25)	0.78 (0.22)	0.79 (0.23)	0.75	0.78	

*P, Pithy Formulas with key-images; K, key-images; S, stroke sequence; the co-variates, using pretests for calculation, were reported in the SPSS software.*

#### Learning Session

To enhance the internal validity of this study, the participants were randomly assigned to one of the two groups. The participants learned a pair on individual computers, displayed by PowerPoint software (Microsoft Office, 2019). The participants were instructed to refrain from any hand movement and to focus on learning materials on the screen without auditory input; they were informed that the display was completely controlled by the computer program with the assistance of the administer.

**FIGURE 1:**
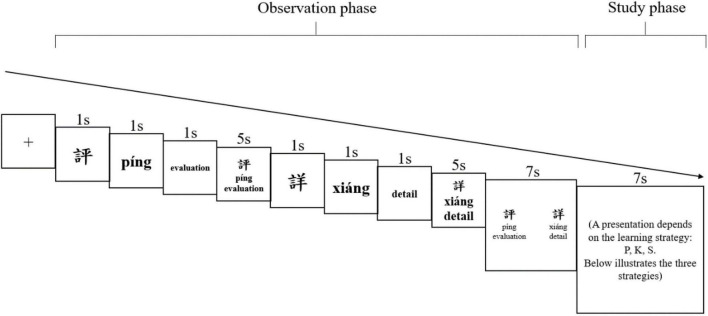
An example of the observation and the study phases for learning character pair 評-詳.

The learning trial for each pair was divided into an observation phase and a study phase. Figure 1 illustrates the trial for each strategy; each trial lasted 30 s and the trial repeated for three times before moving on to the next pair, leading to a 90-s learning duration for each pair. The entire learning session for 15 pairs, including two 1-min breaks, was approximately 23 min.

For each trial, specifically, the observation phase lasted for 23 s. First, an eight-s observation to a character’s form, sound, and meaning was provided by the following sequence of events: a character’s form was shown for 1 s, followed by 1-s *pinyin* display, and then 1-s English translation display; then, the form, *pinyin*, and the English translation were displayed together for 5 s. Second, the next character in the pair was displayed with the same time frames. Next, the participant had a seven-second exposure to the pair’s forms, *pinyin*, and English translations together. Furthermore, proceeding to the study phase that lasted for 7 s, the participant was prompted to use a given strategy to study each pair. Finally, this learning trial for one pair (30 s) repeated for a total of three times before the screen moved on to the next pair.

**FIGURE 2:**
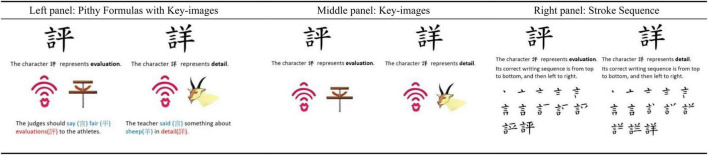
Illustrations showing the three different strategies for learning character pair (in the similar group) 評-詳.

Focusing on the three strategies that differed as to which they promote attention to the form and meaning of characters, Figure 2 presents an illustration for showing the three strategies for learning the same character pair “評-詳” (meaning: evaluation detail; *pinyin*: píng-xiáng). In the Pithy Formulas with a Key-images block, a *form* + *meaning* strategy with visual and verbal codes, key-images were accompanied by a few words, explaining how to integrate key-images to help memorize the form and meaning of the characters. In the Key-images block, a *form* + *meaning* strategy with visual code, the key-images were presented without explanations to the images. In the Stroke Sequence block, a *form-emphasis* strategy, the stroke-by-stroke sequences were accompanied by sentences, explaining the general writing order of the characters. All display was static.

#### Immediate Posttest

After the learning session, the participants were given 5 min to perform a distraction task (i.e., Task Load Index; Hart and Staveland, 1988) to reduce recency effects in testing. Next, the participants completed a writing task and two recognition tasks in the same format as the pretest. After finishing the tasks, the participants responded to a survey, asking about their experiences with the three strategies (see section “Measurement”).

#### Delayed Posttest

One week after the learning session, to assess the maintenance effect of the interventions, the participants were asked to complete the same tests as they did in the pretest and the immediate posttest. Finally, a paper version of the learning materials and a debriefing sheet were given to the participants after the completion of all the tasks.

## Learning and Retention Results

The analysis of covariance (ANCOVA) was adopted to mitigate the possible effect of the participants’ knowledge of learning materials, given that our participants were not novice learners of Chinese (Fisher, 1947). ANCOVA helps to adjust the posttest means for pretest differences among intact groups (Dimitrov and Rumrill, 2003). Thus, for all learning measures, while treating the participants’ pretest as a co-variate, we conducted three-way (3 × 2 × 2) ANCOVAs to examine the effect of learning strategies (Pithy Formulas with Key-images, Key-images, and Stroke Sequence) and presentation of character pairs (similar and dissimilar) over time (immediately after learning and 1 week after learning). Given that the pretest scores would be treated as co-variates in ANCOVA, we also performed *t*-tests on the scores between the pretest and those in the immediate posttest and the delayed posttest for the two groups, respectively (all *p*s < 0.001). As for the learner experience ratings, because this survey aimed to investigate the participants’ experiences, we did not collect pre-learning results as a co-variate. Thus, a one-way repeated ANOVA was adopted to analyze the difference among the three strategies, followed by Bonferroni-corrected paired comparisons when the difference reached significance.

Statistical Package for the Social Science (SPSS) version 23.0 software was used. The significance level was set at α = 0.05, and partial eta-squared ($\eta_{\text{p}}^{2}$) was reported as an effect size; when the difference reached significance, pairwise comparisons with Bonferroni correction were performed to identify the pattern of differences. Table 3 shows descriptive statistics for all learning measures over time.

### Character Writing Task

Both the continuous and the all-or-none scoring revealed that the Pity Formulas with Key-images yielded higher writing accuracies than the other two strategies immediately after learning. Also, the interactions among strategy, group, and testing time varied by scoring schemes.

#### Continuous Scheme (Stroke Scoring)

When scored at the continuous level, the Pithy Formulas with Key-images showed generally better learning outcomes than the Stroke Sequence strategy for both groups in the immediate posttest. The test of regression homogeneity indicated that the slopes did not significantly differ, *F*(2,124) < 1, which qualified the hypothesis of regression homogeneity. Thus, we continued the ANCOVA analysis. The three-way interaction was significant, Strategy × Group × Time, *F*(2,126) = 4.34, *p* = 0.020, $\eta_{\text{p}}^{2}$ = 0.07.

For the group that learned with similar character pairs, in the immediate posttest, higher writing scores were found in the Pithy Formulas with Key-images than those for Key-images (*p* = 0.006) and Stroke Sequence (*p* = 0.002), while the scores for Key-images and Stroke Sequence did not differ (*p* = 1.000). In the delayed posttest, no difference was found among the three strategies (all *p*s > 0.05).

As for the group that learned with dissimilar pairs, in the immediate posttest, Pithy Formulas with Key-images also yielded higher writing scores than Stroke Sequence (*p* = 0.046), while other comparisons were not found (all *p*s > 0.05). In the delayed posttest, there were no differences found for strategy used (all *p*s > 0.05).

In addition to the significant three-way interaction, significant main effects were also found: The Strategy main effect, *F*(2,126) = 4.27, *p* = 0.016, $\eta_{\text{p}}^{2}$ = 0.06, reflected that Pithy Formulas with Key-images led to writing scores significantly higher than that of Stroke Sequence (*p* = 0.001) and marginally higher than that of Key-images (*p* = 0.080), while no difference was found between Key-images and Stroke Sequence (*p* = 0.397). The Group main effect, *F*(1,63) = 4.51, *p* = 0.038, $\eta_{\text{p}}^{2}$ = 0.07, revealed that the dissimilar group outperformed the similar group (*p* = 0.038). The Time main effect, *F*(2,126) = 4.47, *p* = 0.038, $\eta_{\text{p}}^{2}$ = 0.07, showed that all the participants scored higher in the immediate posttest than the delayed posttest (*p* < 0.001). We did not find any two-way interaction: Strategy × Group: *F*(2,126) < 1; Group × Time: *F*(2,126) = 1.51, *p* = 0.224; Strategy × Time: *F*(2,126) = 1.69, *p* = 0.188.

#### All-or-None Scheme (Character Scoring)

When scored at the all-or-none level, Pithy Formulas with Key-images was the most effective strategy for both groups immediately after learning. We used the pretest results as a co-variate. The test of regression homogeneity showed that the slopes did not differ, *F*(2,124) < 1; we continued to perform the ANCOVA analysis. The only statistically significant result was the interaction between Strategy and Time, *F*(2,126) = 4.20, *p* = 0.017, $\eta_{\text{p}}^{2}$ = 0.06. In the immediate posttest, higher writing scores were found in the Pithy Formulas with Key-images than those in the Stroke Sequence (*p* < 0.001) and the Key-images (*p* = 0.021), while the latter two did not differ (*p* = 0.793). None of the differences lasted for the delayed posttest. The following tests did not reach significance: three-way interaction: Strategy × Group × Time, *F*(2,126) < 1; two-way interactions: Strategy × Group, *F*(2,126) < 1, Time × Group, *F*(2,126) = 1.19, *p* = 0.280; and main effects: Strategy, *F*(2,126) = 1.44, *p* = 0.242, Group, *F*(1,63) = 3.67, *p* = 0.060, and Time, *F*(2,126) = 3.73, *p* = 0.058.

### Recognition

#### Chinese-to-English Recognition Task

For accuracy in choosing the correct meaning based on a given character, no significant effect was found. Taking the pretest as a co-variate, the test of regression homogeneity showed that the slopes did not differ, *F*(2,124) = 1.67, *p* = 0.192. In the follow-up analysis, we did not find any significance: all *F*s < 1 for Strategy, Group, Time, and Strategy × Group × Time; Strategy × Group: *F*(2,124) = 1.42, *p* = 0.245; Group × Time: *F*(2,124) = 2.30, *p* = 0.134; Strategy × Time, *F*(2,124) = 1.98, *p* = 0.142.

#### English-to-Chinese Recognition Task

For accuracy in identifying correct Chinese characters based on a given English word, scores in the English-to-Chinese task reflected whether the participants can correctly differentiate visually similar characters. Taking the pretest as a co-variate, the test of regression homogeneity showed that the slopes did not differ, *F*(2,124) < 1. In the follow-up analysis, only the main effect of Strategy reached significance, *F*(2,126) = 3.72, *p* = 0.027, $\eta_{\text{p}}^{2}$ = 0.06; but the pairwise comparisons with Bonferroni adjustment did not find any significant difference in any pair of strategies, all *p*s > 0.35. No other significant effect was found: all *F*s < 1 for Strategy × Group × Time, Group × Time, Strategy × Time, Group, Time; Strategy × Group, *F*(2,124) = 2.09, *p* = 0.128.

### Learner Experience Ratings

**FIGURE 3:**
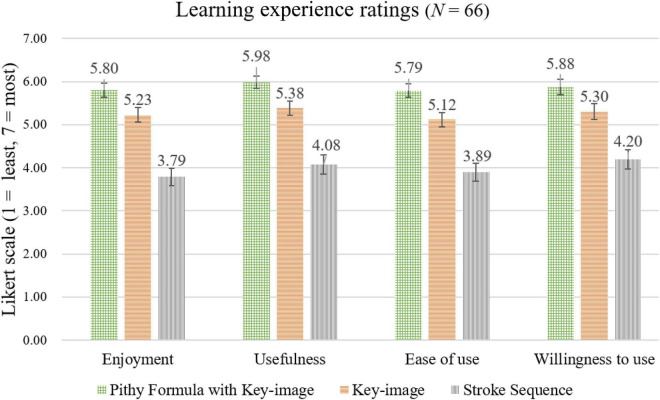
The participants’ mean ratings on enjoyment, usefulness, ease of use, and willingness to use for each learning strategy.

The learners’ ratings of their experiences with each learning strategy were made on four seven-point Likert scales. The top of the scale (7) was the maximum positive response on enjoyment, usefulness, ease of use, and willingness to use. Figure 3 shows the mean ratings for these scales. The participants gave the highest ratings to the Pithy Formulas with Key-images on each scale. Strategy effects were significant for each scale: Enjoyment, *F*(2,130) = 29.65, *p* < 0.001, $\eta_{\text{p}}^{2}$ = 0.313; Usefulness, *F*(2,130) = 25.81, *p* < 0.001, $\eta_{\text{p}}^{2}$ = 0.284; Ease of use, *F*(2,130) = 26.92, *p* < 0.001, $\eta_{\text{p}}^{2}$ = 0.293; Willingness to use, *F*(2,130) = 16.66, *p* < 0.001, $\eta_{\text{p}}^{2}$ = 0.204. Both the Pithy Formulas with Key-images and the Key-images strategies were rated more enjoyable than the Stroke Sequence strategy (all *p*s < 0.001), and the Pithy Formulas with Key-images was marginally more enjoyable than the Key-images (*p* = 0.078). The participants rated the Pithy Formulas with the Key-images more useful than the Key-images (*p* = 0.045) and the Stroke Sequence (*p* < 0.001), and the Key-image more useful than the Stroke Sequence (*p* < 0.001). Similarly, for the ease of use, the Pithy Formulas with the Key-images was rated easier to use than the Key-images (*p* = 0.016) and the Stroke Sequence (*p* < 0.001), and the Key-images easier than the Stroke Sequence (*p* < 0.001). Finally, for willingness to use, the Stroke Sequence was rated lower than the Key-images (*p* < 0.001) and the Pithy Formulas with the Key-images (*p* = 0.003), while no difference was found between the Key-images and the Pithy Formulas with the Key-images (*p* = 0.106).

## Discussion

This study supported non-beginning CSL learners to build robust orthographic representations in Chinese by addressing the challenges of learning visually similar characters. The learning intervention was conducted *via* a combination of three learning strategies (Pithy Formulas with Key-images, Key-images, and Stroke Sequence, differing in the extent to which they promote interrelated attention to the form and meaning of characters) and two methods of presentation (visually similar vs. dissimilar pairs). In the pretest–posttest equivalent-group design, the reading and writing performances of 66 participants were measured immediately after learning and 1 week after for retention. The key findings were that (1) immediately after learning, the Pithy Formulas with the Key-images yielded the highest levels of learning in writing characters, regardless of the method of presentation; (2) when presenting learning targets with visually similar pairs, the Pithy Formulas with the Key-images outperformed the Key-images and the Stroke Sequence in the immediate writing; (3) the learners experience ratings favored the two strategies involving the Key-images over the Stroke Sequence. Previous studies examining the effect of the Key-images usually had characters presented in a dissimilar fashion and reported that the Key-images outperformed Stroke Sequence (e.g., Chang et al., 2019). Our findings in the similar group showed the effectiveness of integrating the Key-images and the Pithy Formulas (a *form* + *meaning* strategy combining visual and verbal codes) over the Key-images (a *form* + *meaning* strategy utilizing visual code) and the Stroke Sequence (a *form-emphasis* strategy), suggesting greater applications of the Key-images in supporting visually similar character learning.

The discussion is organized as follows: first, we discussed the impact of learning strategy for reading and writing as well as the learners’ experience ratings (see section “Learning Strategy of Pithy Formulas With Key-Images Supports Character Writing and Positive Learning Experiences”). Next, we explored the effects of material presentation and focused on its interaction with learning strategy under the theoretical design principles of strategies (see section “The Superiority of the Pithy Formulas With the Key-Images Over the Key-Images and the Stroke Sequence Depends on Material Presentation”). Finally, we discussed research limitation and future directions (see section “Research Limitations and Future Directions”) and then offered an overall conclusion (see section “Conclusion”).

### Learning Strategy of Pithy Formulas With Key-Images Supports Character Writing and Positive Learning Experiences

For reading, regardless of Chinese to English or English to Chinese, all three strategies were effective for our participants. They learned the associations between form and meaning, and they were able to correctly distinguish one character from the others. This finding is in line with prior research, showing that adult non-beginning CFL learners quickly pick up the perceived patterns of characters (Xu et al., 2013). In contrast, for writing, it was a productive, more challenging task for the participants, requiring them to access form-meaning links, retrieve accurate orthographic features, and reproduce whole characters. Prior research on character learning, regardless of being conducted with L1 (e.g., Tan et al., 2005; Bi et al., 2009) or L2 (Chang et al., 2014) learners of Chinese, usually considered writing accuracy as an indicator of robust learning on orthographic representations. On this interpretation, the observation that our participants’ writing differed by learning strategy suggests that the character writing task provides more information than the reading tasks to investigate the intervention. Thus, we continued our discussion based on the results found in character writing.

In discussing the main effect of learning strategy immediately after learning, which shows that the Pithy Formulas with Key-images lead to the highest accuracy rates in writing, we revisited its design principles based on the Elaboration theory (Reigeluth, 1999) and the Dual-coding theory (Paivio, 1986, 2006). Specifically, the Pithy Formulas with the Key-images had the participants develop meaningful imagery-verbal elaboration for linking together the meaning of a character and its constituent components. This design largely instantiated the Elaboration theory (Reigeluth and Stein, 1983; Reigeluth, 1999) by organizing learning materials into meaning context. Moreover, the Pithy Formulas with the Key-images afforded verbal code (i.e., explanation sentences) to provide the participants with context to memorize the combination of components, and this functioned together with the visual code (i.e., Key-images). Such effects are in line with character learning research, applying the Dual-coding theory (e.g., Kuo and Hooper, 2004). That is, the synergy of verbal and visual codes in the Pithy Formulas with the Key-images enhanced memory consolidation and further stabilized memory traces in character learning. Thus, with meaning-prompted cues, reproducing character forms from memory can be largely supported with dual codes.

An alternative explanation for the strategy effect observed in the immediate posttest is learners’ motivation. The learner experience ratings (see section “Learner Experience Ratings”) showed that the learners consistently expressed positive opinions (i.e., enjoyment, usefulness, ease of use, and willingness to use in the future) on the *form* + *meaning* strategies over the *form-emphasis* strategy. Specifically, the Pithy Formulas with the Key-images was rated higher than the Key-images, which, in turn, was rated higher than the Stroke Sequence on the scales of usefulness and ease of use. These positive opinions on the Pithy Formulas with the Key-images possibly promoted the learners’ motivation; therefore, using this strategy, they exhibited higher writing accuracy in the immediate posttest. This alternative explanation was in line with prior research (e.g., Tyng et al., 2017), suggesting that positive emotion facilitates learning and memory processes. While the subjective difference indicated in the experience ratings was larger than the accuracy difference indicated in writing immediately after learning, this view for interpreting the strategy effect echoed previous studies (Chou, 2009a; Tsai et al., 2021), which proposed to integrate both subjective (e.g., motivational and affective aspects) and objective factors (e.g., strategy) in investigating the process of character learning.

### The Superiority of the Pithy Formulas With the Key-Images Over the Key-Images and the Stroke Sequence Depends on Material Presentation

Next, we explored the effects of material presentation. In the context of learning to read across writing systems, echoing literature in learning distinctive features of English letters (e.g., Samuels, 1969; Williams and Ackerman, 1971), our findings on learning Chinese characters may not be an ideal comparison because the formations of graphs with visual features are highly contrastive (Verhoeven and Perfetti, 2021). However, the theory of perceptual learning (Gibson, 1969) has been applied across orthographies (Gibson and Levin, 1975). This theory postulates that learning is to extract meaningful information through higher order relations among features of objects and events. In our case of presenting objects (e.g., target characters), the events (e.g., visually similar pairs vs. dissimilar pairs) possessed different higher order relations, and the results showed that the dissimilar group outperformed the similar group in writing immediately after learning. Thus, we speculated that, in forming the higher-order relations, the dissimilar pairs might provide more distinct visual features than the similar pairs so that the participants who learned with similar pairs recalled character forms easier while writing. However, given that the advantage of dissimilar pairs did not last long, this speculation merited further investigation.

Within the Chinese writing system, prior research on learning visually similar characters was relatively scarce. Also, these studies revealed discrepancy between L1 school-aged learners with different levels of achievement in Chinese (Lin, 1997, 1998). Investigating first graders’ character writing (*n* = 148), Lin (1997) reported that learning visually similar characters simultaneously led to worse writing than learning dissimilar characters for high/mid-achievement learners and floor effects for low-achievement learners. However, in the follow-up study (Lin, 1998), the author reported that underachieving learners (*n* = 17) benefited from presenting graphemically similar characters in recognition. The author argued that the process in learning visually similar characters may be mediated by learners’ proficiency and further interacted with measurement difficulty. The present study is the first attempt to investigate how the method of material presentation may affect learning visually similar characters for CSL learners. Although our findings were similar to the previous results of high/mid-range-achievement L1 learners (Lin, 1997), showing that the group that learned with dissimilar pairs outperformed the group that learned with similar pairs in character writing immediately after learning, we interpreted these findings with caution. Specifically, while having a similar pretest–posttest design with previous studies (Lin, 1997, 1998), our participants were adult second-language learners, having greater linguistic and cognitive maturity, and the amount of target characters used in our study was double compared to that of Lin (1997, 1998).

Furthermore, depending on the two methods of material presentation, we discussed their interaction effects with three learning strategies for learning visually similar characters under the design principles of learning strategies. Theoretically, our findings supported the Dual-coding theory (Paivio, 2006) and the Elaboration theory (Reigeluth, 1999). Echoing the Dual-coding theory, the advantage of the Pithy Formulas with the Key-images (combining visual and verbal codes) over the Key-images (only visual code) was specific to the visually similar group. Meanwhile, in the similar group, the Key-images did not surpass the Stroke Sequence. It was possible that, as targets in the similar pairs were too similar to distinguish one from the other, observing sequences of strokes might cause learners to confuse the construction of form presentations. As for the dissimilar group, the findings confirmed the effectiveness of learning strategies involving the Key-images, which were in line with prior research reporting that the Key-images outperformed the Stroke Sequence (Chang et al., 2019; Lin et al., 2021). In accordance with the Elaboration Theory (Reigeluth, 1999), moreover, these findings demonstrated that learning with *form* + *meaning* strategies yielded better recall than the *form-emphasis* strategy.

The superiority of the Pithy Formulas with the Key-images echoed the third stage of the three-stage character-based instructional framework (Chen et al., 2012) – the deliberately-designed Key-images and pithy formulas are effective in supporting learners to efficiently acquire visually similar compounds. Compounds make up over 90% of commonly used characters (Shen, 2005). As proficiency improves, learners of Chinese encounter many compounds, which, in turn, gradually increases their radical awareness (Shen and Ke, 2007). Previous studies have shown that decomposing characters into chunks (i.e., components) and memorizing the chunks together facilitate character learning, retention, and even generalization (e.g., Lam, 2011; Chen et al., 2013). In this study, the Key-images strategy had done so by co-occurring the Key-images that were visually similar to constituent components of each character and associable to their meanings. It could be possible that CSL/CFL learners generalize their learning with the Key-images and the Pithy Formulas to guess the meanings of novel characters, while this plausibility would require further investigation. At least in this present work, the Pithy Formulas with the Key-images has gone one step further, as indicated by the highest writing accuracy immediately after learning, to leverage the Key-images by adding brief but meaningful sentences to elaborate the imagery-verbal link and further strengthen memory.

### Research Limitations and Future Directions

Notwithstanding the effects of intervention in supporting compound character learning, several limitations of the present study must be acknowledged, and the following are our suggestions for future research. First, we are mindful of the fact that the maintenance of strategy effects did not last long. The robustness of each strategy effect merits further investigation. To deal with this issue, we suggest to vary testing times (simultaneous vs. successive) and to provide multiple practice or review opportunities to find an optimal schedule (Koedinger et al., 2012). Second, to secure the internal validity of research, we conducted this study in laboratories with rigorous control, which reduced the external validity of research. To address this trade-off in research design, our next step would be carrying out an *in vivo* study. To examine whether our findings can be generalized to real-life settings, we envisioned that compounds would be taught together with vocabulary, grammar, and lessons that train listening, speaking, reading, and writing skills. This procedure followed prior research, which tested the effect of Key-images on logographic character learning in classrooms (Lin et al., 2021; Tsai et al., 2021) after validating its effectiveness in laboratories (Chang et al., 2019). Third, for material selection, we limited target stimuli to compounds based on visual similarity while excluding logographic characters. Our findings may not be applicable to integral characters, which are also visually alike (e.g., 由田甲申 or 己已巳). Thus, examining the effects of learning strategies on different types of characters is necessary. Lastly, for non-beginning learners of Chinese, it was difficult to control their proficiency. Although we adopted research design (i.e., randomly allocated participants to two groups) and statistical control (i.e., ANCOVA), attempting to enhance internal validity of the present work, the results may have been easier to interpret if future research tests participants were from the same language background, the same age groups, the same proficiency levels, and even identical Chinese learning experiences (e.g., instructors and Chinese language textbooks).

### Conclusion

This study examined the synergetic effects of learning strategies and methods of presenting materials on reading and writing visually similar characters for non-beginning CSL learners. The takeaway points are twofold. For the cognitive aspect, the learning strategies emphasizing both the verbal and visual codes (i.e., the Pithy Formulas with Key-images) outperformed the visual imagery (i.e., the Key-images), which, in turn, surpassed the Stroke Sequence in writing characters immediately after learning. For the affective aspect, CSL learners’ experiences consistently revealed that *form* + *meaning* strategies are more enjoyable, useful, easy to use, and likely to be used in the future than the *form-emphasis* Stroke Sequence. In sum, this study demonstrates a positive effect of *form* + *meaning* mnemonics, arising from the Dual-coding theory and the Elaboration theory, on enhancing learning efficiency and affection for CSL learners. Pedagogical implications of the findings on the broader topic of learning Chinese as a second/foreign language include, but are not limited to, strategy-based Chinese language education, the compilation of textbooks and exam papers, and error detection in CSL/CFL learners’ compositions.

## Data Availability Statement

The raw data supporting the conclusions of this article will be made available by the authors, without undue reservation, to any qualified researcher.

## Ethics Statement

The studies involving human participants were reviewed and approved by Research Ethics Review Committee of National Taiwan Normal University (NTNU). The participants provided their written informed consent to participate in this study.

## Author Contributions

L-YC and Y-YT: conception and design of the study and data analysis and interpretation. Y-YT, C-YL, and L-YC: data collection. L-YC and C-YL: manuscript writing. H-CC: providing supervision and instructions during the whole process. All authors contributed to the article and approved the submitted version.

## Conflict of Interest

The authors declare that the research was conducted in the absence of any commercial or financial relationships that could be construed as a potential conflict of interest.

## Publisher’s Note

All claims expressed in this article are solely those of the authors and do not necessarily represent those of their affiliated organizations, or those of the publisher, the editors and the reviewers. Any product that may be evaluated in this article, or claim that may be made by its manufacturer, is not guaranteed or endorsed by the publisher.

## References

[B1] Adolph K. E. Kretch K. S. (2015). “Gibson’s theory of perceptual learning,” in *International Encyclopedia of the Social & BehavioralSciences*, 2nd Edn, ed. Wright J. D. (Amsterdam: Elsevier Inc), 127–134. 10.3758/bf03207081

[B2] American Council on the Teaching of Foreign Languages (2012). *ACTFL ProvisionalProficiencyGuidelines.* Yonkers, NY: ACTFL.

[B3] Bi Y. Han Z. Zhang Y. (2009). Reading does not depend on writing, even in Chinese. *Neuropsychologia* 47 1193–1199. 10.1016/j.neuropsychologia.2008.11.006 19056407

[B4] Brysbaert M. New B. (2009). Moving beyond Kucera and Francis: a critical evaluation of current word frequency norms and the introduction of a new and improved word frequency measure for American English. *Behav. Res. Methods* 41 488–496. 10.3758/BRM.41.4.977 19897807

[B5] Chang L. Y. Chen J. Y. Perfetti C. A. Chen H. C. (2019). The effect of key-image mnemonics to support character learning of Chinese-as-foreign-language learners. *J. Chin. Lang. Teach.* 16 31–74.

[B6] Chang L. Y. Chen Y. C. Perfetti C. A. (2018). GraphCom: a multidimensional measure of graphic complexity applied to 131 written languages. *Behav. Res. Methods* 50 427–449. 10.3758/s13428-017-0881-y 28425059

[B7] Chang L. Y. Xu Y. Perfetti C. A. Zhang J. Chen H. C. (2014). Supporting orthographic learning at the beginning stage of learning to read Chinese as a second language. *Int. J. Disabil. Dev. Educ.* 61 289–306.

[B8] Chen H. C. Chang L. Y. Chiou Y. S. Sung Y. T. Chang K. E. (2011). Chinese orthography database and its application in teaching Chinese characters. *Bull. Educ. Psychol.* 43 269–290.

[B9] Chen H. C. Chen H. C. Chang T. H. (2012). *The Establishment, Extension, and Application of the Chinese Orthographic and Error-Type Databases. Retrieved from National Taiwan Normal University Aim for the Top University Project.* Taipei: National Taiwan Normal University.

[B10] Chen H. C. Hsu C. C. Chang L. Y. Lin Y. C. Chang K. E. Sung Y. T. (2013). Using a radical-derived character e-learning platform to increase learner knowledge of Chinese characters. *Lang. Learn. Technol.* 17 89–106.

[B11] Chen H. C. Lin Z. X. (2015a). *Learning Chinese Characters with Drawing I.* Taipei: Cheng Chung Book Co., Ltd.

[B12] Chen H. C. Lin Z. X. (2015b). *Learning Chinese Characters with Drawing II.* Taipei: Cheng Chung Book Co., Ltd.

[B13] Chen H. C. Lin Z. X. Chang L. Y. (2021). *Learning Chinese Characters with Drawing III.* Taipei: Cheng Chung Book Co., Ltd.

[B14] Chou P. H. (2009a). Study of Chinese similar form of the character teaching by theory of learning. *J. Natl. U. Univ.* 6 79–98. 10.29847/JNUU.200906.0005

[B15] Chou P. H. (2009b). The introduction of principles of learning vocabulary by visualization. *J. Natl Taichung Univ. Hum. Arts* 23 55–68. 10.7037/JNTUHA.200906.0055

[B16] Clark J. M. Paivio A. (1991). Dual coding theory and education. *Educ. Psychol. Rev.* 3 149–210. 10.1007/bf01320076

[B17] Davis F. D. (1989). Perceived usefulness, perceived ease of use, and user acceptance of information technology. *MIS Q.* 13 319–340. 10.2307/249008

[B18] De Vellis R. F. (2003). *Scale Development: Theory and Applications.* Thousand Oaks, CA: Sage Publications.

[B19] DeFrancis J. (1989). *Visible Speech: The Diverse Oneness of Writing Systems.* Honolulu, HI: University of Hawaii Press.

[B20] Dimitrov D. M. Rumrill P. D. Jr. (2003). Pretest-posttest designs and measurement of change. *Work* 20 159–165. 12671209

[B21] Faul F. Erdfelder E. Buchner A. Lang A.-G. (2009). ). Statistical power analyses using G*Power 3.1: tests for correlation and regression analyses. *Behav. Res. Methods* 41 1149–1160. 10.3758/BRM.41.4.1149 19897823

[B22] Fisher R. A. (1947). The analysis of covariance method for the relation between a part and the whole. *Biometrics* 3 65–68. 10.2307/3001641 20255205

[B23] Gan J. (2020). The study on Chinese character acquisition errors of foreign students. *Int. J. Learn. Teach.* 6 38–42. 10.18178/ijlt.6.1.38-42

[B24] Gibson E. J. (1969). *Principles of Perceptual Learning and Development.* New York, NY: Appleton-Century-Crofts.

[B25] Gibson E. J. (1991). “Learning to read,” in *An Odyssey in Learning and Perception*, ed. Gibson E. J. (Cambridge, MA: The MIT Press), 393–412.

[B26] Gibson E. J. Levin H. (1975). *The Psychology of Reading.* Cambridge, MA: The MIT Press.

[B27] Hart S. G. Staveland L. E. (1988). Development of NASA-TLX (Task Load Index): results of empirical and theoretical research. *Adv. Psychol.* 52 139–183. 10.1016/S0166-4115(08)62386-9

[B28] Hirshorn E. A. Harris L. N. (2022). Culture is not destiny, for reading: highlighting variable routes to literacy within writing systems. *Ann. N. Y. Acad. Sci.* 1–17. 10.1111/nyas.14768 [Epub ahead of print]. 35313016

[B29] Ho C. S. H. Ng T. T. Ng W. K. (2003). A “radical” approach to reading development in Chinese: the role of semantic radicals and phonetic radicals. *J. Lit. Res.* 35 849–878. 10.1207/s15548430jlr3503_3

[B30] Ho S. Chow B. Wong S. Waye M. Bishop D. V. M. (2012). The genetic and environmental foundation of the simple view of reading in Chinese. *PLoS One* 7:e47872. 10.1371/journal.pone.0047872 23112862 PMC3480450

[B31] Hoover W. Gough P. (1990). The simple view of reading. *Read. Writ. Interdiscip. J.* 2 127–160.

[B32] Jin H. G. (2006). Multimedia effects and Chinese character processing: an empirical study of CFL learners from three different orthographic backgrounds. *J. Chin. Lang. Teach. Assoc.* 41 35–56.

[B33] Ke C. (1998). Effects of strategies on the learning of Chinese characters among foreign language students. *J. Chin. Lang. Teach. Assoc.* 33 93–112. 10.3389/fpsyg.2017.01846 29109694 PMC5660119

[B34] Koedinger K. R. Corbett A. C. Perfetti C. (2012). The knowledge-learning-instruction framework: bridging the science-practice chasm to enhance robust student learning. *Cogn. Sci.* 36 757–798. 10.1111/j.1551-6709.2012.01245.x 22486653

[B35] Kuo L. J. Kim T. J. Yang X. Li H. Liu Y. Wang H. (2015). Acquisition of Chinese characters: the effects of character properties and individual differences among second language learners. *Front. Psychol.* 6:986. 10.3389/fpsyg.2015.00986 26379562 PMC4550700

[B36] Kuo M. L. Hooper S. (2004). The effects of visual and verbal coding mnemonics on learning Chinese characters in computer-based instruction. *Educ. Technol. Res. Dev.* 52 23–38. 10.1007/bf02504673

[B37] Lam H. C. (2011). A critical analysis of the various ways of teaching Chinese characters. *Elect. J. Foreign Lang. Teach.* 8 57–70. 10.1093/cercor/bhv113 26045566

[B38] Language and Teaching Institute of Beijing Linguistic College (1986). *XiandaiHanyuPinluCidian [Modern Chinese frequency Dictionary].* Beijing: Beijing Language Institute Press.

[B39] Leck K. J. Weekes B. S. Chen M. J. (1995). Visual and phonological pathways to the lexicon: evidence from Chinese readers. *Mem. Cogn.* 23 468–476. 10.3758/bf03197248 7666760

[B40] Levin J. R. (1993). Mnemonic strategies and classroom learning: a twenty-year report card. *Elem. Sch. J.* 94 235–244. 10.1086/461763

[B41] Lin S. J. (1997). A comparison of the ability of first graders in Taiwan to learn graphemically-similar versus graphemically-dissimilar Chinese characters. *Bull. Spec. Educ. Rehabil.* 5 227–251.

[B42] Lin S. J. (1998). A comparison of the ability of first graders with low achievement in Chinese language arts to learn graphemically-similar versus graphemically-dissimilar Chinese characters. *Bull. Spec. Educ. Rehabil.* 6 261–277.

[B43] Lin Z. X. Hsiung H. Y. Lin Y. C. (2021). The research of online key-image strategy on Chinese characters learning effects and preferences to CFL learners. *J. Chin. Lang. Teach.* 18 99–121.

[B44] Liu C. L. Lai M. H. Tien K. W. Chuang Y. H. Wu S. H. Lee C. Y. (2011). Visually and phonologically similar characters in incorrect Chinese words: analysis, identification, and applications. *ACM Trans. Asian Lang. Inform. Proc.* 10 1–39. 10.1145/1967293.1967297

[B45] Liu C. L. Tien K. W. Lai M. H. Chuang Y. H. Wu S. H. (2009). “Capturing errors in written Chinese words,” in *Proceedings of the 47th Annual Meeting of the Association for Computational Linguistics (ACL’09)*, (Singapore), 25–28.

[B46] Ma X. Gong Y. Gao X. Xiang Y. (2017). The teaching of Chinese as a second or foreign language: a systematic review of the literature 2005-2015. *J. Multiling. Multicult. Dev.* 38 815–830. 10.1080/01434632.2016.1268146

[B47] Mohammad A. Ketabi S. (2011). Mnemonic instruction: a way to boost vocabulary learning and recall. *J. Lang. Teach. Res.* 2 178–182. 10.4304/jltr.2.1.178-182

[B48] Nguyen T. P. Zhang J. Li H. Wu X. Cheng Y. (2017). Teaching semantic radicals facilitates inferring new character meaning in sentence reading for nonnative Chinese speakers. *Front. Psychol.* 8:1846. 10.3389/fpsyg.2017.01846 29109694 PMC5660119

[B49] Packard J. (2017). Mnemonics in the Chinese L2 lexicon. *Taiwan J. Chin. Sec. Lang.* 15 53–63.

[B50] Paivio A. (1986). *Mental Representations.* New York, NY: Oxford University Press.

[B51] Paivio A. (1990). *Mental Representations: A Dual Coding Approach*, 2nd Edn. Oxford: Oxford University Press.

[B52] Paivio A. (2006). *Mind and its Evolution: A Dual Coding Theoretical Approach.* Mahwah, NJ: Erlbaum.

[B53] Perfetti C. A. (1999). “Comprehending written language: a blueprint of the reader,” in *The Neurocognition of Language*, eds Brown C. Hagoort P. (Oxford: Oxford University Press), 167–208. 10.1093/acprof:oso/9780198507932.003.0006

[B54] Perfetti C. A. (2007). Reading ability: lexical quality to comprehension. *Sci. Stud. Read.* 11 357–383. 10.1080/10888430701530730

[B55] Perfetti C. A. Dunlap S. (2008). “Learning to read: general principles and writing system variations,” in *Learning to Read Across Languages*, eds Koda K. Zehler A. M. (New York, NY: Routledge), 25–50. 10.4324/9780203935668-9

[B56] Perfetti C. A. Liu Y. Tan L. H. (2005). The lexical constituency model: some implications of research Chinese for general theories of reading. *Psycholog. Rev.* 112 43–59. 10.1037/0033-295X.112.1.43 15631587

[B57] Qin W. Z. (2014). *The Effectiveness of the Rating of Similarity Degree and Creative Radical Elaborate Mnemonics in Distinguishing the Chinese Character Pairs of Similar Patterns. Unpublished master’s thesis.* Taipei: National Taiwan Normal University.

[B58] Reigeluth C. Stein F. (1983). “The elaboration theory of instruction,” in *Instructional Design Theories and Models*, ed. Reigeluth C. (Hillsdale, NJ: Erlbaum Associates).

[B59] Reigeluth C. M. (1999). “The elaboration theory: Guidance for scope and sequences decisions,” in *Instructional-Design Theories and Models: A New Paradigm Of Instructional Theory*, Vol. Volume II ed. Reigeluth R. M. (Mahwah, NJ: Lawrence Erlbaum Associates), 425–454.

[B60] Samuels S. J. (1969). Effect of simultaneous versus successive discrimination training on paired-associate learning. *J. Educ. Psychol.* 60 46–48. 10.1037/h0026671

[B61] Shen H. H. (2005). An investigation of Chinese-character learning strategies among non-native speakers of Chinese. *System* 33 49–68. 10.1016/j.system.2004.11.001

[B62] Shen H. H. (2010). Imagery and verbal coding approaches in Chinese vocabulary instruction. *Lang. Teach. Res.* 14 485–499. 10.1177/1362168810375370

[B63] Shen H. H. (2013). Chinese L2 literacy development: cognitive characteristics, learning strategies, and pedagogical interventions. *Lang. Linguist. Compass* 7 371–387. 10.1111/lnc3.12034

[B64] Shen H. H. Ke C. (2007). Radical awareness and word acquisition among nonnative learners of Chinese. *Mod. Lang. J.* 91 97–111. 10.1111/j.1540-4781.2007.00511.x

[B65] Shu H. Chen X. Anderson R. C. Wu N. Xuan Y. (2003). Properties of school Chinese: implications for learning to read. *Child Dev.* 74 27–47. 10.1111/1467-8624.00519 12625434

[B66] Sung K. (2012). A study on Chinese-character learning strategies and character learning performance among American learners of Chinese. *Chin. Sec. Lang. Res.* 1 193–210. 10.1515/caslar-2012-0012

[B67] Taft M. Chung K. (1999). Using radicals in teaching Chinese characters to second language learners. *Psychologia* 42 243–251.

[B68] Tan L. H. Spinks J. A. Eden G. F. Perfetti C. A. Siok W. T. (2005). Reading depends on writing, in Chinese. *Proc. Natl. Acad. Sci.* 102 8781–8785. 10.1073/pnas.0503523102 15939871 PMC1150863

[B69] Teng S. H. Tome M. Yeh H. H. Wu J. H. Guan Y. (2008). “The Chinese character teaching grammar and Chinese Character error corpus for chinese learners,” in *Proceedings of 2008 International Annual Conference of Teaching Chinese as a Second Language*, ed. Teng S. H. 278–290, Miaoli.

[B70] Tokowicz N. Michael E. Kroll J. F. (2004). The roles of study abroad experience and working memory capacity in the types of errors made during translation. *Biling. Lang. Cogn.* 7 255–272. 10.1017/s1366728904001634

[B71] Tsai M. H. Chang L. Y. Chen H. C. Lin C. L. (2021). Effects of key-image mnemonics on Chinese instruction for first-grade students’ achievement and interest toward Chinese learning. *Int. J. Educ. Res.* 109 1–13. 10.1016/j.ijer.2021.101856

[B72] Tyng C. M. Amin H. U. Saad M. N. M. Malik A. S. (2017). The influences of emotion on learning and memory. *Front. Psychol.* 8:1454. 10.3389/fpsyg.2017.01454 28883804 PMC5573739

[B73] Verhoeven L. Perfetti C. (2021). Universals in learning to read across languages and writing systems. *Sci. Stud. Read.* 26, 1–15. 10.1080/10888438.2021.1938575

[B74] Verhoeven L. van Leeuwe J. (2012). The simple view of second language reading throughout the primary grades. *Read. Writ. Interdiscip. J.* 26 1806–1818. 10.1007/s11145-011-9346-3 22923881 PMC3422459

[B75] Wang L. (2014). The effects of single and dual coded multimedia instructional methods on Chinese character learning. *Chin. Sec. Lang. Res.* 3 1–25. 10.1515/caslar-2014-0001

[B76] Wang Z. G. (2011). *Questions and Answers on Modern Chinese Character.* Beijing: Peking University Press.

[B77] Williams C. (2013). Emerging development of semantic and phonological routes to character decoding in Chinese as a foreign language learners. *Read. Writ.* 26 293–315. 10.1007/s11145-012-9368-5

[B78] Williams J. P. Ackerman M. D. (1971). Simultaneous and successive discrimination of similar letters. *J. Educ. Psychol.* 62 132–137. 10.1080/00223980.1975.9915772 1235793

[B79] Winke P. Abbuhl R. (2007). Taking a closer look at strategies for Chinese language learning. *Foreign Lang. Ann.* 40 697–712. 10.1111/j.1944-9720.2007.tb02888.x

[B80] Xu P. Jen T. (2005). Penless” Chinese language learning: a computer-assisted approach. *J. Chin. Lang. Teach. Assoc.* 40 25–42.

[B81] Xu Y. Chang L. Y. Zhang J. Perfetti C. A. (2013). Reading, writing, and animation in character learning in Chinese as a foreign language. *Foreign Lang. Ann.* 46 423–444. 10.1111/flan.12040

[B82] Yeh S. L. Li J. L. (2002). Role of structure and component in judgments of visual similarity of Chinese characters. *J. Exp. Psychol. Hum. Perfept. Perform.* 38 933–947. 10.1037/0096-1523.28.4.933 12190259

[B83] Yeh S. L. Li J. L. Chen I. P. (1997). The perceptual dimensions underlying the classification of the shapes of Chinese characters. *Chin. J. Psychol.* 39 47–74.

[B84] Yu Z. (2012). Debate on Chinese as one of the most difficult languages in the world. *Lang. Teach. Linguist. Stud.* 4 38–45.

