# Supplementary material for: The Effect of Visual Mnemonics and the Presentation of Character Pairs on Learning Visually Similar Characters for Chinese-As-Second-Language Learners
Source: Front Psychol. 2022 May 9;13:783898. doi: 10.3389/fpsyg.2022.783898 (PMC9125332; doi:10.3389/fpsyg.2022.783898)
Supplement: Supplementary file 1 [file Presentation_1.pdf]

Appendix 1. Pithy Formulas with Key-images for 30 learning targets (presented in 15 visually similar pairs)

Character	Pithy formula	Key-images	Character	Pithy formula	Key-images
埋	The land (土) miles (里) away is the place where I buried (埋) my dog.	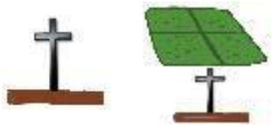 土 里	理	Kingdom borders run many miles (里), and it's the King's (王) job to manage (理) their integrity.	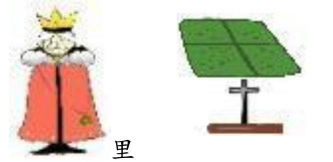 王 里
責	Protecting the shell (貝) for your host (主) is your responsibility (責).	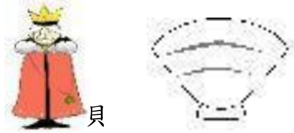 主 貝	貢	People devote tools (工) and shells (貝) as the King's tribute (貢).	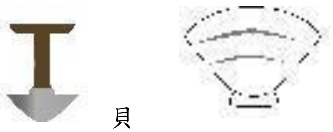 工 貝
書	In ancient times (日), we used brushes (聿) to write words in the books (書).	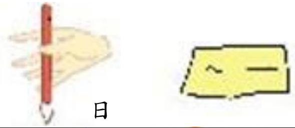 聿 日	畫	We used brushes (聿) to draw fields (田) and rivers on the painting (畫).	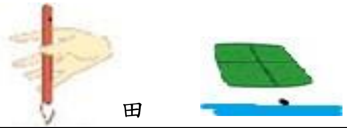 聿 田
稚	The one who played with birds (隹) in the grain (禾) is a young (稚) kid.	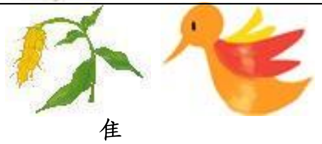 禾 隹	椎	The bird (隹) in the tree (木) is a vertebrate (椎).	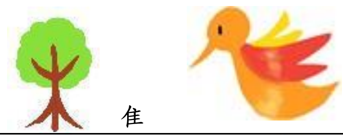 木 隹
計	If you say (言) ten (十) ideas, the ten ideas become a plan (計).	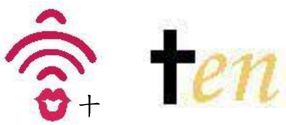 言 十	討	Please say (言) the size (寸) of your body when you have a discussion (討) with me.	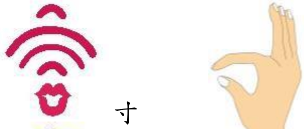 言 寸
塊	Ghosts (鬼) will dwell on land (土), especially the pieces (塊) beside tombs.	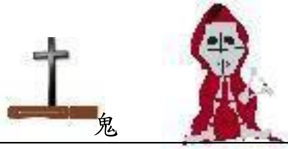 土 鬼	瑰	The king (王) freaked out when he saw ghosts (鬼) holding roses (瑰).	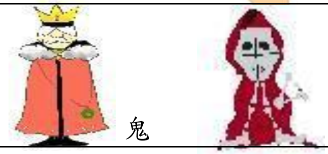 王 鬼
買	It costs me four (四) shells (貝) to buy (買) a cup.	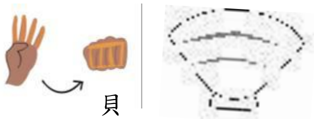 四 貝	賈	The man selling shells (貝) in the west (西) is a merchant (賈).	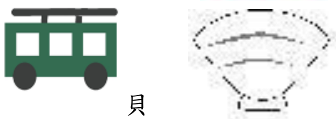 西 貝
問	He is opening his mouth (口) at the door (門), waiting to ask (問) something.	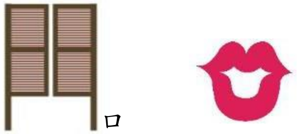 門 口	間	The sun (日) shines through the door (門) between (間) 11 o'clock and 12 o'clock.	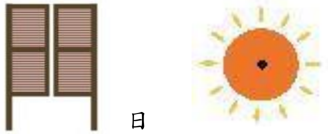 門 日

Character	Pithy formula	Key-images	Character	Pithy formula	Key-images
棵	The tree (木) covered with fruit (果) is the one (棵) that I wish to have in my house.	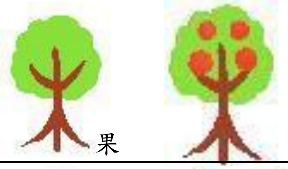	稞	The Han Chinese eat grain (禾) and fruits (果), while the Tibetans eat hulless barley (稞).	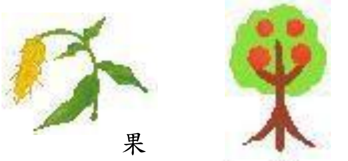
評	The judges should say (言) fair (平) evaluations (評) to the athletes.	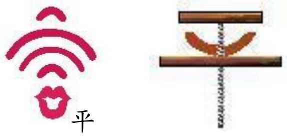	詳	The teacher said (言) something about sheep (羊) in detail (詳).	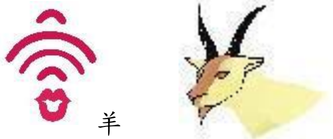
忡	The boy standing in the middle (中) of road made our hearts (心) full of distress (忡).	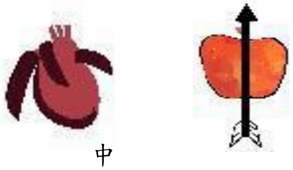	怏	Not putting me in your center (央) makes my heart (心) feel discontented (怏).	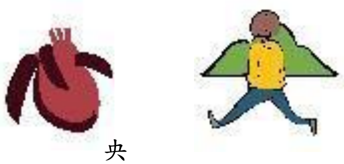
查	You can avoid the dazzling of daybreak (旦) by staying under the tree (木) to check (查) your bag.	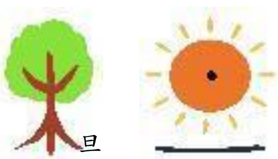	杳	After the sun (日) goes down, the trees (木) are obscure (杳).	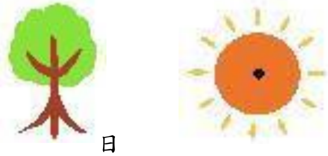
鈦	The gold (金) bracelet is too (太) expensive, so I bought a titanium (鈦) one instead.	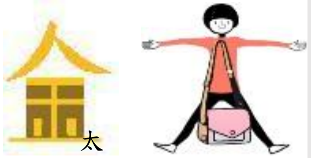	鈸	You can pull out (犮) the golden (金) cymbals (鈸) from the jazz drums.	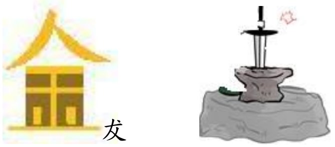
話	The words (言) spoken through the movement of the tongue (舌) become the spoken language (話).	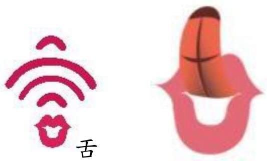	詁	使用現代話語說明古書意義是訓詁<br>Using modern words (言) to explain the meaning of ancient (古) books is exegesis (詁).	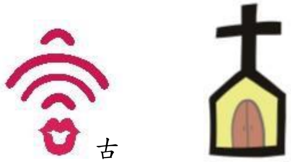
季	The child (子) hidden in the grain (禾) is happy no matter which season (季) it is.	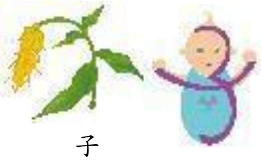	李	My son (子) sat under the tree (木) full of plums (李).	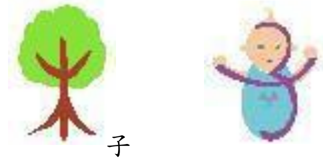
