# Supplementary material for: The Effect of Visual Mnemonics and the Presentation of Character Pairs on Learning Visually Similar Characters for Chinese-As-Second-Language Learners
Source: Front Psychol. 2022 May 9;13:783898. doi: 10.3389/fpsyg.2022.783898 (PMC9125332; doi:10.3389/fpsyg.2022.783898)
Supplement: Supplementary file 2 [file Presentation_2.pdf]

Appendix 2. Examples and instructions for three learning measures

Measure	Instruction to participants	Example provided	Knowledge assessed	Format
Character writing	Please write down the character in the blank that matches the English definition.	star<br>( )	Visual-orthographic form representation (productive)	Paper-and-pencil
	There are two sessions in the task. One is reading the English meaning of a word and choosing its correct Chinese character (English-to-Chinese), and the other is reading a Chinese character and choosing its correct English meaning (Chinese-to-English).		Visual-orthographic form representation (receptive)	
Recognition: Chinese-to-English	Please choose the English definition that best matches the character. Each question had four options, and only one was the correct answer.	波<br>( ) Ⓐwave Ⓑslope Ⓒsea Ⓓbrain	Form-meaning link, by assessing learners' ability to identify a given character's meaning from three distractors	Computerized
Recognition: English-to-Chinese	Please choose the Chinese character that best matches the English definition. Each question had four options, and only one was the correct answer.	power<br>( ) Ⓐ力 Ⓑ刀 Ⓒ刃 Ⓓ忍	Form-meaning link, by assessing learners' ability to differentiate one character from its visually similar counterpart based on a given meaning	Computerized
